# Relationship between ventilator bundle compliance and the occurrence of ventilator-associated events: a prospective cohort study

**DOI:** 10.1186/s12912-022-00997-w

**Published:** 2022-08-01

**Authors:** Eman Arafa Hassan, Suad Elsayed abdelmotalb Elsaman

**Affiliations:** Critical Care and Emergency Nursing Department, Faculty of Nursing, Alexandria University, Alexandria, Egypt

**Keywords:** Ventilator-associated pneumonia, Ventilator-associated events, Ventilator bundle, Compliance

## Abstract

**Background:**

Instead of ventilator-associated pneumonia (VAP), the modern definition of ventilator-associated events (VAEs) has been introduced to identify infectious and noninfectious respiratory complications. Some studies revealed that compliance to the ventilator bundle is associated with decreased occurrence of VAP, but little is known about its association with the decrease of VAEs occurrence.

**Methods:**

A prospective cohort research design was used. Data were collected over eight months from May 2019 to February 2020 in five general intensive care units. The researchers assessed the compliance to ventilator care bundle using the Institute for Healthcare Improvement ventilation bundle checklist. Mechanically ventilated patients were prospectively assessed for the occurrence of VAEs using a pre-validated calculator from the Centers for Disease Control and Prevention. All are non-invasive tools and no intervention was done by the authors.

**Results:**

A total of 141 mechanically ventilated patients completed the study. The odds ratio of having VAEs in patients who received ventilator bundle was -1.19 (95% CI, -2.01 to -0.38), a statistically significant effect, Wald χ2(1) = 8.18, *p* = 0.004.

**Conclusion/ implications for practice:**

Ventilator bundle compliance was associated with a reduced risk for VAEs occurrence. Nurses should comply with the ventilator bundle because it is associated with decreased VAEs occurrence.

## Background

Ventilator-associated events (VAEs) are particular complications of mechanical ventilation that develop after 48 h from initiating mechanical ventilation [1]. It has three definitions which are ventilator-associated conditions (VAC), infection-related ventilator-associated complications (IVAC), and possible ventilator-associated pneumonia (PVAP) [2]. The VAC detects respiratory deterioration following at least two days of ventilator setting stability or improvement. An IVAC is a concurrent inflammatory change and treatment course adjustment in a patient with a VAC. The last of the VAE is PVAP. It is a subset of IVAC with positive respiratory cultures [2, 3].

The Centers for Disease Control and Prevention (CDC) recommended shifting from the VAP definitions to VAEs definitions in 2013. The reason is the high clinician variability in evaluating VAP which increases the subjectivity in VAP surveillance and hinders comparison between institutional VAP rates and benchmarks. Many commonly used VAP definitions have the disadvantage of requiring radiographic evidence of pneumonia. According to the evidence, chest radiograph findings do not accurately identify VAP [3, 4].

The use of evidence-based guidelines in VAEs prevention decreases harm and improves patient safety and quality of care. Ventilator bundle is one of the evidence-based practices that may decrease the risk for VAEs [5]. The Institute for Healthcare Improvement (IHI) recommended the application of the ventilator bundle elements. The ventilator bundle has five elements which are head of the bed elevation 35 to 45 degrees, sedation vacation and assess readiness to extubate, peptic ulcer prophylaxis, deep vein thrombosis (DVT), and use of chlorhexidine for oral care [6].

Studies aimed to prevent VAEs evaluated one or more elements of the ventilator bundle [7, 8], but little is known about the application of the five elements of the bundle together. Compliance with ventilator bundle and prevention of VAEs are collaborative responsibilities between physicians and nurses in intensive care units. So, the current study aimed to identify the relationship between ventilator bundle compliance and the occurrence of VAEs.

## Methods

### Design

A prospective cohort research design was used.

### Setting

This study was conducted in five general ICUs at Alexandria Main University Hospital. The total number of beds in these units is 58 beds.

### Participants

G* Power version 3.1 was used to calculate patient sample size [9]. Based on a medium effect size of 0.5, power = 0.80, and alpha = 0.05, the required sample size is 132 patients. 20% of the sample was added to overcome the attrition rate [10].

Inclusion criteria were adult mechanically ventilated critically ill patients. Exclusion criteria were patients attached to invasive MV for less than 3 days, and patients contraindicated to any component of the ventilator care bundle application. They are patients with (1) spinal cord injury, (2) any type of shock, (3) allergy to chlorhexidine, (4) refractory hypoxia, (5) high intracranial pressure, and (6) coagulation problems.

### Data tools

Two tools were used to collect data for this study. Tool one is the ventilator care bundle compliance checklist. It is a pre-validated ventilation bundle checklist from the Institute for Healthcare Improvement [11]. The checklist has five elements: (1) head of bed elevation at 30–45°, (2) daily sedation interruptions and daily assessment of readiness to extubate, (3) peptic ulcer prophylaxis, (4) DVT prophylaxis, and (5) daily oral care with chlorhexidine. Each element was scored as either one for compliant (yes), or zero for noncompliant (no) [12].

Tool two is the ventilator-associated events calculator. It is a pre-validated calculator from the CDC (2020). A calculator is a web-based tool on the CDC website, version 7.0. This calculator is used to identify the clinical criteria of the VAEs. It includes three levels: VAC, IVAC, and PVAP. The minimum PEEP (cmH2O) and minimum FiO2 are the data required to calculate the VAC. Body temperature, WBC count, and antibiotic use are data that calculate IVAC. The data required to calculate PVAP are the characteristics of the tracheal aspirate specimen culture.

### Data collection

Data were collected by the researchers for approximately eight months from May 2019 to February 2020. All patients attached to MV at zero-day of initiating mechanical ventilation were assessed against the inclusion and exclusion criteria. Patients who met the inclusion criteria were included in the study. Patients who were weaned from the mechanical ventilator before reaching the third day of mechanical ventilation were excluded from the study because VAEs cannot be assessed as an outcome of the application of a ventilator bundle.

Health care providers’ compliance with all elements of the ventilator bundle was assessed every day for two weeks. During the morning, evening, and night shifts, the ventilator bundle compliance was checked. If one element of the bundle was not done or done incorrectly at any shift of the day, it was recorded as non-compliant. If all elements of the ventilator bundle were done correctly during the whole day, it was recorded as compliant.

The VAEs were assessed daily from the third day of mechanical ventilation until two weeks based on VAEs online calculator version 7.0 [4]. The output of the calculator is no VAEs or the presence of VAEs which has three ordinal categories: VAC, IVAC, and PVAP. The data which were required fed the online calculator are ventilator FiO2, PEEP, patients' temperature, WBCs counts, new antibiotic starting, and the respiratory culture result.

Variables associated with VAEs in previous literature were assessed for each patient [10, 13]. These factors include age, gender, history of smoking, diagnosis, level of consciousness, and invasive operations.

### Statistical analysis

Data were analyzed using SPSS software package version 25.0 (IBM Corp., Armonk, NY). Continuous variables were represented as mean with standard deviation, whereas categorical variables as number with percentage. The Shapiro–Wilk test was used to check the normality of continuous variables. Ordinal logistic regression was used to identify variables related to the occurrence of VAEs. Monto Carlo test was used to compare between VAEs occurrence in patients who received and patients who did not receive ventilator care bundle. The level of statistical significance was set at *p*-value ≤ 0.05.

### Administrative and ethical considerations

Before collecting data, approval from the Research Ethics Committee, Faculty of Nursing, Alexandria University, Egypt was obtained. We obtained informed consent from patients or their legal guardians before their participation. Official permission was obtained from the administrative authorities of the Alexandria Main University Hospital after revision and approval of the research methods. No experimental protocols or invasive procedures were used in the current study. We used non-invasive tools that are already recommended by the international guidelines of the CDC for routine use in intensive care units. The anonymity and the privacy of the patients, as well as the confidentiality of the collected data, were assured. All methods were carried out in accordance with relevant guidelines and regulations.

## Results

**Fig. 1:**
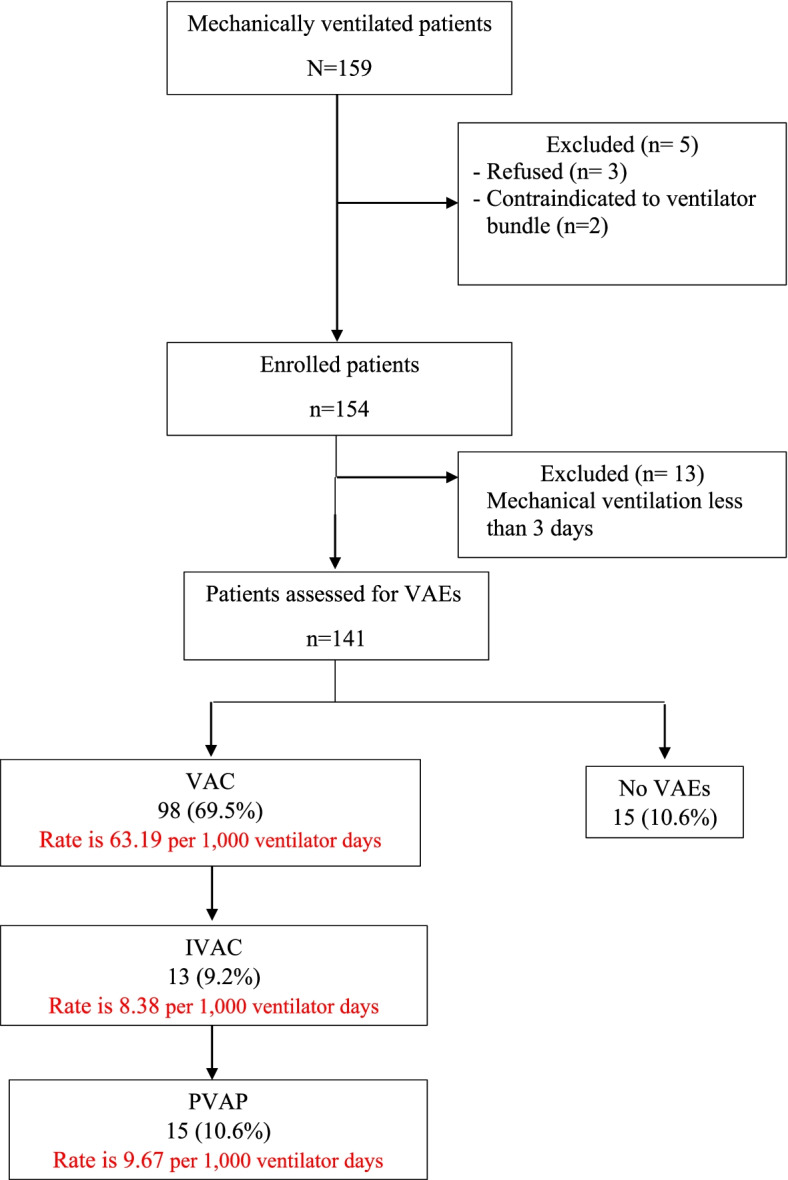
Flowchart.; VAEs, ventilator-associated events; VAC, ventilator-associated condition; IVAC, infection-related ventilator-associated complication; PVAP, possible ventilator-associated pneumonia

During the study period, 159 patients were assessed for meeting the inclusion criteria. A total of 141 patients met the inclusion criteria and completed the study. A total of 18 patients dropped out of the study. Three of them refused to participate in the study, two of them were contraindicated to head of bed elevation because of shock state, and 13 of them had mechanical ventilation for less than three days. Evaluation of patients who completed the study according to the VAEs criteria revealed that 10.6% of them had no VAEs, 69.5% of them had VAC, 9.2% of them had IVAC, and 10.6% of the patients had PVAP as shown in Fig. 1. Also, it can be noted from the figure that the VAC rate was 63.19 per 1,000 ventilator days, while the IVAC rate was 8.38 per 1,000 ventilator days. PVAP was found to be 9.67 per 1,000 ventilator days.

Table 1 illustrates the patients’ demographic and clinical data. The patients’ mean age was 48 ± 19.5 years and 70.2% of the patients were males. The highest frequent diagnosis was respiratory disorders (38.3%), and 54.6% of the patients were smokers. The frequency of patients with a decreased level of consciousness was 37.6%. Mean.

**Table 1:** Patients’ demographic and clinical data (*n* = 141)

Patients' data	N (%) or M ± SD
**Age**	48 ± 19.5
**Gender**	
Male	99 (70.2%)
Female	42 (29.8%)
**History of smoking**	
No	64 (45.4%)
Yes	77 (54.6%)
**Diagnosis**	
Respiratory	54 (38.3%)
Cardiovascular	30 (21.3%)
Neurological	44 (31.2%)
Others	13 (9.2%)
**Invasive operation**	
No	137 (97.2%)
Yes	4 (2.8%)
**Decreased level of consciousness**	
No	88 (62.4%)
Yes	53 (37.6%)
**Duration of mechanical ventilation**	11 ± 4

Ordinal logistic regression in Table 2 shows that the odds ratio of having VAEs in patients who received ventilator care bundle was -1.19 (95% CI, -2.01 to -0.38), a statistically significant effect, Wald χ2(1) = 8.18, *p* = 0.004. On contrary, patients with multiple diagnoses had a significant increase in their odds (2.90 with 95% CI from 1.00 to 4.81) to have VAEs.

**Table 2:** Ordinal logistic regression for variables related to the occurrence of VAEs

Variables	OR	SE	Wald	df	*P* value	95% Confidence Interval	
						**Lower Bound**	**Upper Bound**
Age	0.01	0.02	0.09	1	.771	-0.03	0.04
Gender	-0.59	0.80	0.55	1	.460	-2.16	0.98
History of smoking	0.96	0.79	1.47	1	.226	-0.59	2.50
Decreased level of conscious	1.50	0.47	10.44	1	.001^*^	0.59	2.42
Multiple diagnoses	2.90	0.97	8.94	1	.003^*^	1.00	4.81
Invasive operation	0.40	1.12	0.13	1	.719	-1.79	2.60
Ventilator bundle compliance	-1.19	0.42	8.18	1	.004^*^	-2.01	-0.38

^*^ Statistically significant at *p* ≤ 0.05; *OR* Odds ratio, *SE* Standard error, *df* Degree of freedom

Table 3 shows a statistically significant difference (*p* = 0.002) between VAEs occurrence in patients who received and patients who did not receive ventilator bundle. The frequency of patients who are free from VAEs was 15.1% in patients who received ventilator bundle, while it was 2.1% in patients who did not receive ventilator bundle. Also, frequencies of IVAC and PVAP were lower in patients who received ventilator bundle (5.4%, and 7.5%) than those who did not (16.7%, and 16.7%). In contrast with other VAEs types, the frequency of VAC was higher in patients who received ventilator care bundle (72.0%) than in those who did not receive ventilator care bundle (64.6%).

**Table 3:** Difference between VAEs occurrence in patients who received and patients who did not receive ventilator bundle

Type of VAEs	Ventilator bundle compliance				X^2^ (P^MC^)
	**Yes**		**No**		
	**n**	**%**	**n**	**%**	
No VAEs	14	15.1%	1	2.1%	X^2^ = 12.12 P^MC^ = .002*
VAC	67	72.0%	31	64.6%	
IVAC	5	5.4%	8	16.7%	
PVAP	7	7.5%	8	16.7%	
Total	93	100%	48	100%	

*VAEs* Ventilator-associated events, *VAC* Ventilator associated condition, *IVAC* Infection related ventilator associated condition, *PVAP* Possible ventilator associated pneumonia, *MC* Monto Carlo test

^*^ Statistically significant at *p* ≤ 0.05

## Discussion

The current study evaluated the relationship between ventilator bundle compliance and the occurrence of VAEs. It was found that compliance with ventilator bundle was associated with lower VAEs occurrence. Specific to components of VAEs, it was found that application of ventilator bundle was associated with lowered both IVAC and PVAP, however, it did not associate with lower VAC.

The current study results are in congruency with the previous studies which evaluated the relation between the application of ventilator bundle elements and the occurrence of VAEs [14, 15]. Evidence shows that the application of multidisciplinary interrelated interventions focused on VAEs prevention dramatically decreases VAEs rates [16]. This may be the rational that the ventilator bundle was associated with decreased VAEs occurrence. In this study ventilation bundle compliance was measured using the ‘all or none method. Nurses in critical care settings work collaboratively with other health care providers to apply all elements of the bundle as recommended by the IHI [16, 17].

The literature implies that components of the ventilator bundle are linked to lower VAE rates; however, most of these studies correlate between one of the bundle components and the rate of VAEs [1, 14, 15]. One study evaluated only sedation interruption and breathing trial on the occurrence of VAEs, and it was associated with lower VAE rates [7]. Oral care also was found to reduce VAEs [18]. Another study evaluated several evidence-based practices such as minimizing sedation, paired daily spontaneous awakening and breathing trials, early mobility, conservative fluid management, conservative transfusion thresholds, and low tidal volume ventilation which also were associated with lower VAEs rates [8].

The rate of VAEs varies across different countries [19, 20]. The VAE incidence in the United States is between 2–11.79/1000 ventilator days [20]. Another study suggests that almost half of the adult ventilated patients develop any type of VAE during the first 30 days of mechanical ventilation [19]. Specific to components of VAEs, A surveillance in France found that 77% of mechanically ventilated patients had at least one VAC, and 29% of patients had one infection-related VAEs [21]. In the current study VAC rate was 69.5% (63.19 per 1,000 ventilator days), while infection-related VAEs rate was 19.8% (18.05 per 1,000 ventilator days).

High rates of VAC were observed in several studies, despite the application of ventilator bundle [21–23]. One of these studies confirmed our study finding that ventilator bundle elements, including semi-recumbent positioning, oral care with chlorhexidine, venous thromboembolism prophylaxis, stress ulcer prophylaxis, daily spontaneous breathing trials, and sedative interruptions, were not associated with VAC occurrence [22].

The VAEs algorithm includes an ordinal set of definitions designed to detect both infectious and noninfectious complications as well as direct and indirect consequences of mechanical ventilation [3]. This may be the reason that the ventilator bundle did not associate with the reduced occurrence of VAC in the current study. The ventilator bundle aims to prevent aspiration, decrease colonization in the upper respiratory tract and decrease the length of mechanical ventilation [17]. That is why it was associated with the decrease of both IVAC and PVAP, but VAC has multifactorial non-infectious risk factors that cannot be controlled by only ventilator bundle [24].

A study suggested that mandatory modes of ventilation and positive fluid balance are risk factors for VAC [22]. Another study suggested that the main causes of VAC are the noninfective events of atelectasis, acute respiratory distress syndrome, pulmonary edema, and pulmonary embolism [25]. Also, this study confirms that the ventilator bundle is associated with less incidence of infectious ventilator events but they fail to reduce the rates of VAC [25]. So, further studies are needed to find nursing and multidisciplinary practices that are associated with lower VAC rates.

### Implication

Nurses and other health care providers should comply with the ventilator bundle because it is associated with decreased VAEs occurrence.

### Limitations

This study is a prospective observational study that could be replicated by experimental study design to assess the effect of ventilator bundle on the prevention of VAEs. Another limitation of this study is the collection of data from only one hospital. Also, the relatively small sample size is another limitation.

## Conclusion

The ventilator bundle compliance was associated with decreased VAEs occurrence, specifically IVAC and PVAP. Regarding VAC, a multidisciplinary effort should be directed toward reduction of these non-infectious complications of VAEs because the compliance with ventilator bundle was not associated with lower VAC occurrence.

## Data Availability

The data and materials of the current study are not publicly available due to confidentiality reason but are available from the corresponding author on reasonable request.
